# Dowling, P.; et al. Characterization of Contractile Proteins from Skeletal Muscle Using Gel-Based Top-Down Proteomics. *Proteomes* 2019, *7*, 25

**DOI:** 10.3390/proteomes7030028

**Published:** 2019-07-15

**Authors:** Paul Dowling, Margit Zweyer, Dieter Swandulla, Kay Ohlendieck

**Affiliations:** 1 Department of Biology, Maynooth University, National University of Ireland, Maynooth, W23F2H6 Co. Kildare, Ireland; 2 MU Human Health Research Institute, Maynooth University, Maynooth, W23F2H6 Co. Kildare, Ireland; 3 Institute of Physiology II, University of Bonn, D-53115 Bonn, Germany

The authors wish to make the following correction to their paper [1]:

The Figure 2 and Figure 5 have some irregular typesetting. The corrected Figure 2 and Figure 5 should be:

## Figures and Tables

**Figure 2:**
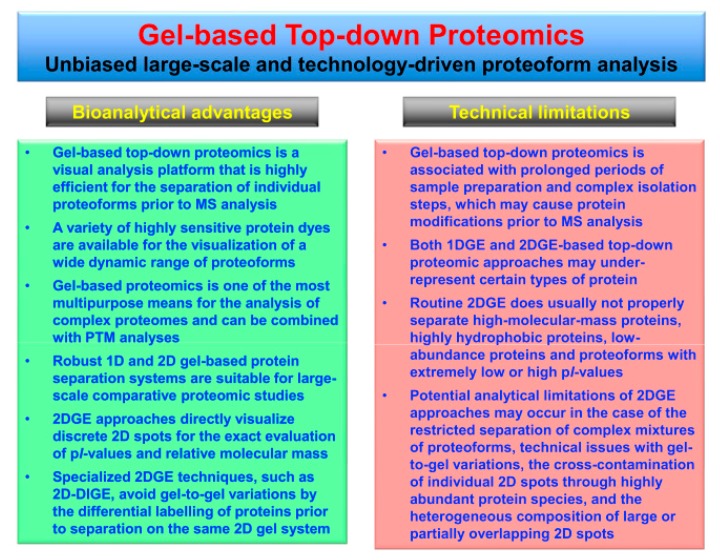
Overview of the bioanalytical advantages versus potential technical limitations of gel-based top-down proteomics, especially in relation to two-dimensional gel electrophoresis (2DGE) approaches.

**Figure 5:**
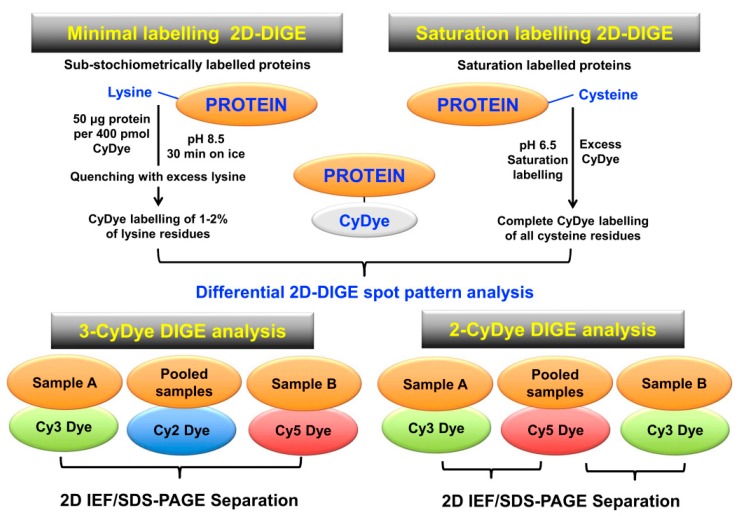
Overview of fluorescence two-dimensional difference in-gel electrophoresis (2D-DIGE) techniques for the comparative analysis of proteoforms. The upper panel shows the two main DIGE approaches for the systematic pre-electrophoretic labelling of protein fractions using fluorescent CyDyes. These methods utilise minimal sub-stochiometric labelling of assessable lysines in proteins or saturation labelling of assessable cysteines in proteins. The lower panel outlines three-dye (Cy2, Cy3, Cy5) versus two-dye (Cy3, Cy5) DIGE labelling methodology.
